# Knowledge, Attitude, and Practices (KAP) Survey among Veterinarians, and Risk Factors Relating to Antimicrobial Use and Treatment Failure in Dairy Herds of India

**DOI:** 10.3390/antibiotics10020216

**Published:** 2021-02-22

**Authors:** Deepthi Vijay, Jasbir Singh Bedi, Pankaj Dhaka, Randhir Singh, Jaswinder Singh, Anil Kumar Arora, Jatinder Paul Singh Gill

**Affiliations:** 1 School of Public Health and Zoonoses, College of Veterinary Science, Guru Angad Dev Veterinary and Animal Sciences University, Ludhiana 141004, India; deepthivijay@kvasu.ac.in (D.V.); pankaj.dhaka2@gmail.com (P.D.); sainirandhir74@gmail.com (R.S.); gilljps@gmail.com (J.P.S.G.); 2 Department of Veterinary and Animal Husbandry Extension Education, College of Veterinary Science, Guru Angad Dev Veterinary and Animal Sciences University, Ludhiana 141004, India; jaswindervet@rediffmail.com; 3 Department of Veterinary Microbiology, College of Veterinary Science, Guru Angad Dev Veterinary and Animal Sciences University, Ludhiana 141004, India; aroraak65@gmail.com

**Keywords:** antimicrobial resistance, antimicrobial usage, bovine, India, KAP survey, veterinarians

## Abstract

The indiscriminate usage of antimicrobials in the animal health sector contributes immensely to antimicrobial resistance (AMR). The present study aims to assess the antimicrobial usage pattern and risk factors for AMR in animal husbandry sector of India. A cross-sectional survey about Knowledge, Attitude, and Practices (KAP) among veterinarians was carried out using a questionnaire comprising of 52 parameters associated with antibiotic use and the emergence of AMR in dairy herds. Respondents’ KAP scores were estimated to rank their level of knowledge, attitude, and practice. Furthermore, risk factors associated with treatment failure were analyzed by univariable and multivariable analyses. Out of a total of 466 respondents, the majority had average knowledge (69.5%), neutral attitude (93.2%), and moderate practice (51.3%) scores toward judicious antibiotic usage. Veterinarians reported mastitis (88.0%), reproductive disorders (76.6%), and hemoprotozoan infections (49.6%) as the top three disease conditions that require antibiotic usage. Most of the veterinarians (90.6%) resorted to their “own experience” as the main criteria for antibiotic choice. The use of the highest priority critically important antimicrobials (HPCIA) listed by the World Health Organization (WHO) in animals, particularly quinolones (76.8%) and third-generation cephalosporins (47.8%), has been reported. On multivariable regression analysis of the risk factors, the lack of cooperation of the dairy farmers in the completion of a prescribed antibiotic course by the veterinarian and the demand for antibiotic use even in conditions not requiring antibiotic use were found to be significantly associated with the outcome variable “treatment failure” having respective odds of 1.8 (95%CI: 1.1–3.0) and 3.6 (95%CI: 2.3–5.8) (*p* < 0.05). The average KAP score of veterinarians, poor farm management practices, lack of awareness among farmers on prudent antibiotic use, and lack of antibiotic stewardship are the significant factors that need attention to combat the rising AMR in veterinary sector in India.

## 1. Introduction

Antimicrobial resistance is one of the greatest public health threats that has been projected to cause globally 10 million deaths and US$100 trillion economic loss by 2050 [1]. In order to meet the food security of burgeoning human population, the economic scale production of food animals favor the high-density farming operations, which could double the antibiotic consumption by livestock in developing countries by 2030 [2,3]. The widespread application of antibiotics to food animal populations imposes strong selection pressure, which contributes to the emergence, spread, and persistence of resistant pathogens to other animals, humans, and the environment [4]. The awareness on antibiotic resistance in human medicine has gained momentum; however, the role of animal husbandry practices in tackling antibiotic resistance are still being discussed with limited awareness among stakeholders, especially in developing countries [5]. 

India is bestowed with huge livestock wealth comprising of 193.5 million cattle and 109.9 million buffaloes [6]. The emerging intensive farming practices of the country has been posited as the hotspots of antibiotic resistance, and by 2030, the use of antibiotics in food animals has been projected to increase by 82% [2]. The threat of antibiotic resistance from the foods of animal origin has been discussed in many recent studies in India, highlighting the need for the judicious use of antibiotics in the animal health sector of the country [7,8,9]. Albeit, a “National Action Plan on Antimicrobial Resistance” has been enforced for optimizing antibiotic use in the country, strict enforcement still needs to be executed at the ground level [10,11,12].

While the reliability of data on the usage of antibiotics in the animal husbandry sector is questioned in general, some developing countries including India have a negligible amount of data [2,13]. In the midst of antimicrobial resistance crises with limited existing treatment options, mitigation strategies mainly revolve around awareness and proper stewardship for antibiotic usage among the key stakeholders. Thereby, understanding of knowledge, attitude, and practices (KAP) among the main stakeholders (e.g., veterinarians) with regard to antimicrobial use and resistance can help in the development of tailored intervention strategies to address poor practices, lack of knowledge, and negative attitude. Keeping in view the fact that there is no systematic KAP study along with prevailing antibiotic usage patterns and resistance in animal husbandry sector in Indian settings, the objectives of the present study were to assess Knowledge, Attitude, and Practices (KAP) among veterinarians in relevant to antimicrobial usage in animal husbandry sector through cross-sectional surveys, and identify the risk factors for the development of antimicrobial resistance (AMR) in India.

## 2. Material and Methods

### 2.1. Study Design and Questionnaire Development

The descriptive study was designed as a questionnaire-based cross-sectional analysis among the veterinarians of India during February 2020 to June 2020. A comprehensive review of the literature has been conducted to identify the factors influencing knowledge, attitude, and practices (KAP) on antimicrobial usage and resistance among veterinarians [14,15,16]. The questionnaire design was guided by the results from qualitative interviews and focus group discussions with veterinary academicians and farm animal practitioners of the Guru Angad Dev Veterinary and Animal Sciences University, Ludhiana, India. The questionnaire consisted of close-end questions, Likert scale statements, and open-ended questions exploring the existing knowledge, antimicrobial prescribing behaviors, perceptions on antimicrobial usage, and field practices associated with antimicrobial resistance. In addition, the veterinarian’s recommendations were also requested for suggesting the interventions to combat antimicrobial resistance in the animal husbandry sector.

The questionnaire was divided into five sections: (1) Personal information; (2) Health services; (3) Knowledge, attitude, and practices toward antibiotic use; (4) Knowledge, attitude, and practices toward antimicrobial resistance; and (5) Miscellaneous section covering practices and recommendations for combating antimicrobial resistance.

The preliminary draft of the questionnaire having 58 questions was reviewed by five expert researchers to identify ambiguity and content validity. Later, the questionnaire was piloted among 20 veterinarians to assess its duration, clarity, and sequence. During the processing, six questions were omitted that were inappropriate, resulting in a total of 52 questions in the final questionnaire (Supplementary Material File S1).

### 2.2. Sampling Procedure 

The source population of the present study comprised of registered veterinarians (Veterinary Council of India and/or State Veterinary Council) of India, and the study population included veterinarians who fulfilled the inclusion criteria of being farm animal practitioners. The sample size was calculated using the ‘Raosoft calculator’ (Raosoft: http://www.raosoft.com/samplesize.html?nosurvey). The sample size of 377 was estimated based on 50% response distribution, a 5% margin of error, and a 95% confidence interval. The expected response proportion of 50% was assumed based on the fact that both responses and response rates were completely unknown, since there are no previously published similar studies from India. Thereby, a total of 800 questionnaires were sent to the veterinarians selected through registered emails and/or personal contacts from professional societies and social media groups. The questionnaire was administrated by using the online interface of Google Forms (Google LLC, Mountain View, CA, USA) to the target population, and the survey remained open from May 2020 to June 2020. 

### 2.3. Ethical Statement

The research was conducted in accordance with the Declaration of Helsinki and national standards. All the required ethical considerations have been taken into account. The nature of the study was completely voluntary, and informed consent was obtained from study participants. The details of the participants were anonymous, and data confidentiality was properly maintained. 

### 2.4. Statistical Analysis

The completed questionnaires were manually checked for data quality before coding on Microsoft^®^ Office Excel 2010. The study variables were summarized using proportions for qualitative variables and median and median absolute deviation for quantitative variables. The Likert-scale questions were condensed into two categories for analysis. A scoring system was generated by the subject experts of the University, in which the participants were given a score for knowledge, attitude, and practices based on the number of correct or appropriate responses. The overall score was determined based on the sum of correct answers to the eleven knowledge-based questions, four attitude-based questions, and thirteen practice-based questions. The respondent’s level of knowledge/attitude/practices were categorized as “high/positive/good”, “average/neutral/moderate”, or “low/negative/poor” using the ≥75th percentile, <75th to 25th percentile, and <25th percentile of the individual scores, respectively. The Mann–Whitney U test/Kruskal–Wallis H test were used to determine the relationship between demographic characteristics of the veterinarians and their KAP scores. The correlation among the knowledge, attitude, and practice scores were assessed by the Spearman correlation. A *p*-value of ≤0.05 was interpreted as significant. The logistic regression analysis was performed to estimate predictors for the outcome variable, “frequent treatment failure”. The outcome variable “frequent treatment failure” depicting the failure of response of the animal to the first line of antibiotic treatment by the veterinarians was ascertained from the questionnaire. Various risk factors associated with “frequent treatment failure” were used as predictors determined by univariate odds ratio. The multicollinearity was checked to rule out the relationship amongst the independent variables based on the Variable Inflation Factor value (VIF) calculated in an iterative manner. The associations between the selected variables for multivariable analysis had a VIF of less than 2. The interactions between the predictors were checked and were found to be non-significant. The model was constructed by considering all these explanatory variables using the backward stepwise approach using the Likelihood Ratio Test (LRT). The analyses were conducted using SPSS version 24.0 (SPSS Inc., IBM, Armonk, NY, USA).

## 3. Results

A total of 478 (59.7%) responses were received out a total of 800 questionnaires, of which 466 were with complete information. The questionnaires that contained incomplete (*n* = 7) and vague information (*n* = 5) were excluded from the study.

### 3.1. Demographic Information 

The demographic profile of the participants belonged to twenty-five states of India, which were grouped into six geographical regions (Table 1). Out of 466 participants with a median age of 32 years, 70.0% were males and 30.0% were females. The highest number of respondents belonged to the 30–40 age group (37.5%). It was observed that 48.1% of veterinarians had post-graduate qualifications. Most of the veterinarians (62.9%) had less than 10 years of field experience. The majority of the respondents were working in veterinary hospitals (85.6%), while 14.4% were in veterinary polyclinics that had established laboratory facilities. 

### 3.2. Common Diseases Requiring Antibiotic Usage in Bovines

**Figure 1:**
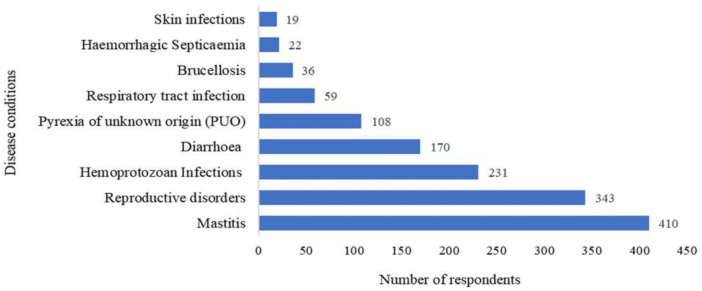
Major disease conditions requiring antibiotic use in bovines in India * (* Question: Top 03 disease conditions that require antibiotic use in bovines. Each veterinarian was asked to choose up to three disease conditions).

Major disease conditions found in bovines requiring antibiotic usage are listed in Figure 1. The veterinarians reported mastitis (*n* = 410), reproductive disorders (*n* = 343), and hemoprotozoan infections (*n* = 231) as the top three disease conditions in bovines where antibiotics are widely used. 

### 3.3. Antibiotic Prescribing Decisions

**Figure 2:**
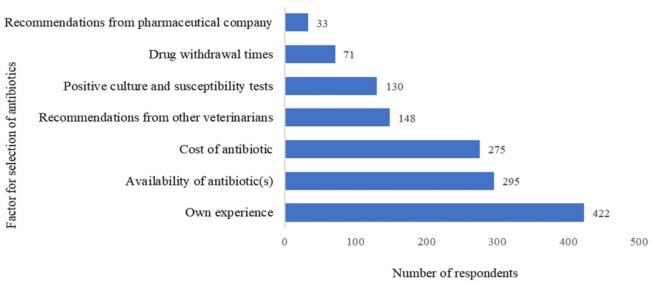
Factors determining the choice of antimicrobial use by veterinarians * (* Question: What are the top three factors in determining the choice of antibiotics use in your treatment? Choose among the following (Own experience/Availability of antibiotic(s)/Recommendations from other veterinarians/Cost of antibiotic/Positive culture and susceptibility tests/Drug withdrawal times/Recommendations from pharmaceutical company). Each veterinarian was asked to choose up to three top factors determining the choice of antimicrobials).

The decision over the choice of antibiotics in various diseases/conditions in bovines was influenced by different factors (Figure 2). The majority of the veterinarians (90.6%) depended on their own experience as the top criteria for choosing antibiotics followed by the availability (63.3%) and cost (59.0%) of the antibiotic. Recommendations from other veterinarians (31.8%) and pharmaceutical companies (7.1%) also influenced their decision regarding antibiotic use. Around 28% of the veterinarians took into account positive culture and sensitivity test results, whereas the withdrawal period of the drug influenced only 15% of veterinarians in prescribing the antibiotics. In addition, 24.9% veterinarians reported that the demand and expectation of farmers influences the prescription behaviors of antibiotics, even for conditions that do not require their use. 

**Figure 3:**
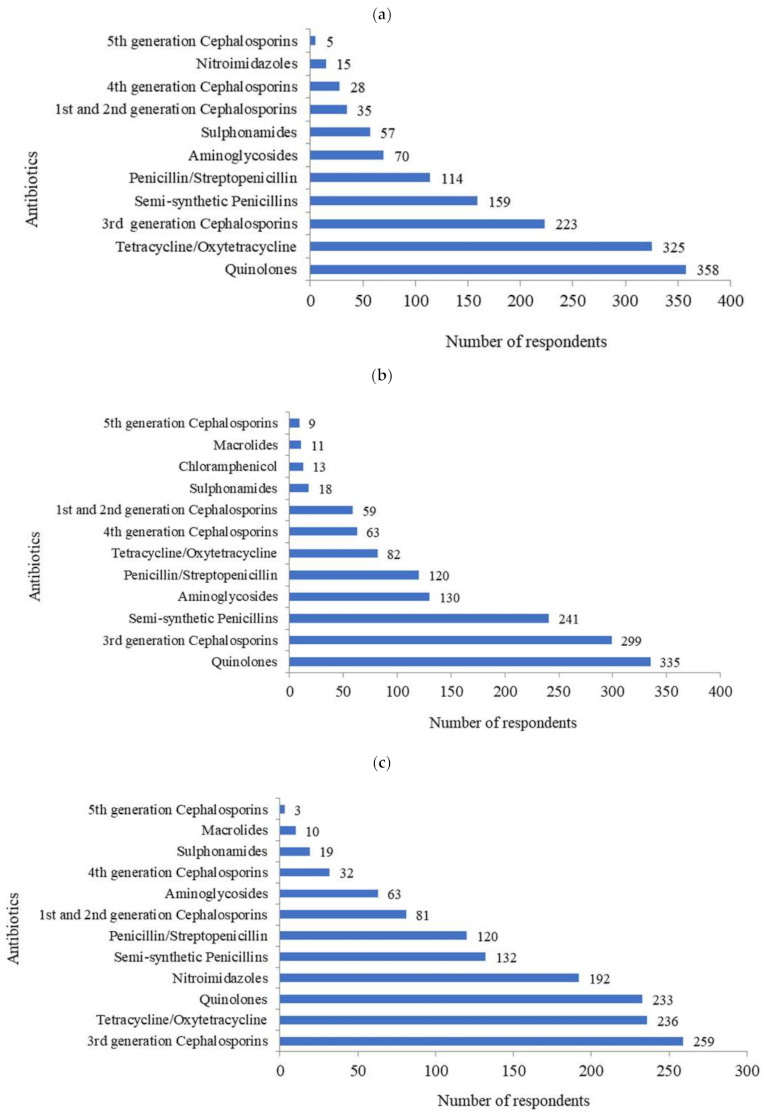
Commonly used antibiotics in bovines ((**a**) overall use; (**b**) use in mastitis; (**c**) use in metritis)* (* Questions: (**a**) Top three frequently used antibiotics in the treatment of bovines; (**b**) Top three frequently used antibiotics for the treatment of mastitis in bovines; (**c**) Top three frequently used antibiotics for the treatment of metritis in bovines. Each veterinarian was asked to choose up to three most commonly used antibiotics).

The veterinarians reported the use of “highest priority critically important antimicrobials” (HPCIA) mentioned by World Health Organization (WHO) [17] in their choices for treatment, *viz.*, quinolones (76.8%; *n* = 358), third-generation cephalosporins (47.8%; *n* = 223), and fourth-generation cephalosporins (6.0%; *n* = 28) (Figure 3a). The quinolones (71.9%, *n* = 335) were the most commonly prescribed antibiotic for mastitis followed by third-generation cephalosporins (64.2%; *n* = 299) (Figure 3b). In case of metritis, third-generation cephalosporins (55.6%; *n* = 259) followed by tetracycline (50.6%; *n* = 236) and quinolones (50.0%; *n* = 233) were the top three commonly used antibiotics (Figure 3c). 

However, 45.5% of the veterinarians were aware of the ‘critically important list of antimicrobials’ of the WHO [17], while 59.2% opined that restriction on the WHO suggested ‘priority antibiotics for human-use only’ is not possible in veterinary therapeutics. The antibiotics in the ‘reserve group’ as proposed by the WHO [18], particularly fourth-generation cephalosporins, were used by 13.5% of the veterinarians in mastitis and by 6.9% of veterinarians in metritis. Moreover, 1.9% of the veterinarians reported the use of fifth-generation cephalosporins in mastitis. In addition, uses of alternate therapies such as herbal medicines were reported by 74.0% veterinarians, whereas 67.2% used probiotics, 43.8% used homeopathic medicine, and 2.4% used indigenous remedies for different disease conditions.

### 3.4. Knowledge, Attitude, and Practice (KAP) Analysis

**Figure 4:**
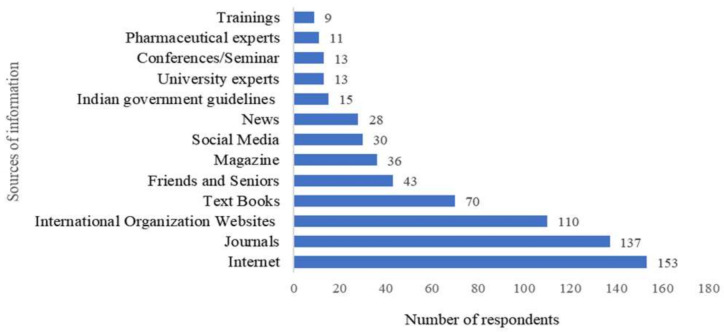
Information sources referred by veterinarians on antibiotic use and resistance * (* Question: What are the major information sources that you refer regularly to for knowledge on antibiotic use and resistance? (The question was open-ended with the provision to answer more than one source of information)).

The knowledge of the respondents on antimicrobial use and resistance was assessed by scoring eleven questions, with the score 1 given to correct answer while 0 was given to incorrect or not sure response (Table 2; Supplementary Material Table S1). The knowledge was scaled as high with a score ≥ 9, average with a score 6–9, and low with a score < 6. The median knowledge score of the respondents was 8.0 ± 1.0. Only 14.2% of the respondents had a high knowledge score, whereas most respondents (69.5%) had an average knowledge score. The majority of the respondents (73%) were regularly updating themselves on antimicrobial resistance, where the internet was the most common information source (Figure 4). A significantly higher knowledge score was observed among the veterinarians who regularly updated themselves compared with those who did not (U statistic: 4.6, *p*-value: 0.00). 

Attitude toward antibiotic use and associated resistance was assessed by four questions (Table 2, Supplementary Material Table S1) with score of 1 for correct, 0.5 for partially correct, and 0 for incorrect or not sure response. The attitude score of ≥2.5 was classified as positive, 0.5–2.5 was classified as neutral and < 0.5 was classified as negative. The majority of the respondents (93.3%) had attitude score in the neutral range, with an overall median of 1.5 ± 0.5. 

The practice scores were assessed for thirteen questions (Table 2; Supplementary Material Table S1) with a score of 1 for correct, 0.5 for partially correct and 0 for incorrect practice. The practice scale with a score of ≥7.5 was classified as good, 4.5–7.5 was classified as moderate and <4.5 was classified as poor. The respondents had a median practice score of 6.0 ± 1.5. The majority of the respondents (51.3%) had a moderate practice score and 27.7% stated poor practice toward antimicrobial usage. In addition, 27.2% of veterinarians had attended training programs on antibiotic usage and resistance. The veterinarians who attended the training program had significantly higher practice scores (U statistic: 5.3, *p*-value: 0.00) and knowledge scores (U statistic: 3.8, *p*-value: 0.00).

### 3.5. Association of KAP Scores with Demographic Characteristics

The association of demographic characteristics and KAP scores were analyzed using the Mann–Whitney U test/Kruskal–Wallis H test (Table 3). A significant difference was observed among the age groups, with higher knowledge (*H* statistic: 10.9, df: 4, *p*-value: 0.03) score in the <30-year age group. Post hoc analysis revealed that the knowledge scores of veterinarians having age <30 differed significantly from the other age groups. The veterinarians with PhD degrees had significantly higher knowledge scores (*H* statistic: 37.8, df: 2, *p*-value: 0.00), and on post hoc analysis, the knowledge scores of all the groups having different educational qualifications differed significantly from each other. Moreover, a higher knowledge (*H* statistic: 19.1, df: 3, *p*-value: 0.00) score was observed among veterinarians having less than 10 years of experience, and post hoc analysis revealed that the knowledge score of veterinarians having less than 10 years of experience and veterinarians with 20–30 years of experience differed significantly from the knowledge score of veterinarians with 30–40 years of experience. The knowledge and attitude scores had no significant difference between the regions, while a higher practice score was observed amongst the veterinarians from the Western region (*H* statistic: 13.7, df: 5, *p*-value: 0.02), and post hoc analysis revealed that the practice score of veterinarians of the Western region differed significantly from that of respondents of the Northern and Eastern region. The veterinarians working in veterinary polyclinics had higher knowledge scores than those working in veterinary hospitals (*U* statistic: 2.2, *p*-value: 0.03).

### 3.6. Correlation between Knowledge, Attitude and Practice Scores

The present study revealed weak linear correlations between knowledge–attitude (*r*  =  0.23, *p*  <  0.000), knowledge–practice (*r*  =  0.20, *p*  <  0.000), and attitude–practice (*r*  =  0.18, *p*  <  0.001) as per the criteria by Cohen (2013) (0–0.25  =  weak correlation, 0.25–0.5 = fair correlation, 0.5–0.75 = good correlation, and >0.75 = excellent correlation) [19].

### 3.7. Risk Factors Associated with Treatment Failure

Most of the veterinarians (86.0%) admitted about ongoing antibiotic abuse in therapeutics, and 98.7% considered antimicrobial resistance as a serious public health issue. Frequent treatment failure has been reported by 21.7% of veterinarians, and therapeutic failure has been observed in mastitis treatment against HPCIA such as quinolones (13.5%), third-generation cephalosporins (11.4%), and high-priority antimicrobials such as synthetic penicillin (11.6%), penicillin (11.4%), and aminoglycosides (9.2%). For metritis treatment, veterinarians reported therapeutic failure against quinolones (2.4%), tetracyclines (2.1%), synthetic penicillins (1.9%), and third-generation cephalosporins (1.7%). The failure of effective therapeutic response to antimicrobials other than antibiotics was reported by 66.1% of veterinarians for antiparasitic drugs and 9.4% for antifungal drugs.

**Figure 5:**
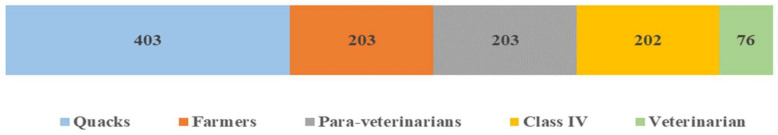
Personnel responsible for irrational use of antibiotics in field *. (Quacks: unauthorized practitioners; Para-veterinarians: diploma holders in Veterinary Science, Class IV: helping staff in veterinary hospitals) (* Question: Whom do you think as responsible for the irrational use of antibiotic in bovines at the field level (select all that apply)? (The question was having the provision to select more than one option)).

The majority of the veterinarians (86.5%) attributed unauthorized practitioners (commonly called “quacks”) followed by farmers and para-vets (43.6% each) as responsible for irrational use of antimicrobials in livestock (Figure 5). The practice of farmers directly acquiring antibiotics from a pharmacy without prescription was reported by 82.8% of the veterinarians, whereas 39.5% of the veterinarians reported non-cooperation of the farmers in the completion of the antibiotic course prescribed by them. However, 31.8% veterinarians organized awareness camps on antibiotic usage and resistance for farmers.

Around 16.3% of veterinarians considered themselves responsible for the injudicious use of antimicrobials, and 39.1% of veterinarians used antibiotics for prophylaxis, especially to prevent outbreaks. The majority of the veterinarians (62.2%) rarely performed antibiotic susceptibility testing to complement their treatment, while 70.6% of veterinarians reported lack of laboratory facilities for performing antibiotic sensitivity testing in/near their hospital. Moreover, only 20.8% veterinarians were aware about the recommendations of the National Antimicrobial Resistance Plan of 2017, India [20].

### 3.8. Univariable and Multivariable Analysis

The univariable analysis for frequent treatment failure associated risk factors pertaining to veterinarian’s and farmer’s practices was carried out by calculating the odds ratio (Table 4). All the variables of univariable analysis were used for building logistic regression models using independent predictors of practices associated with veterinarians and farmers in respect to frequent treatment failure. 

On multivariable logistic regression analysis with a backward stepwise approach using the Likelihood Ratio Test (LRT), the final model contained two variables as depicted in Table 5. With respect to the risk factors associated with veterinarians, “skipping doses of antibiotics” and “allowing farmer to inject subsequent doses of antibiotics after administering first dose of the treatment” were significantly found to be associated with frequent treatment failure, with respective odds ratios of 1.7 (95%CI: 1.1–2.6) and 1.8 (95%CI: 1.1–2.8) (*p*-value: <0.05) (Table 5a). The adjusted odds ratio of “illegitimate demands of farmers for antibiotic use” and “farmer’s non-cooperation in completion of antibiotic course” were found to be significantly associated with “frequent treatment failure”, with respective odds ratios of 3.6 (95%CI: 2.3–5.8) and 1.8 (95%CI: 1.1–3.0) (*p*-value: <0.05) (Table 5b). The Hosmer–Lemeshow test for goodness of fit was found to be non-significant for both the models of veterinarians and farmers (Table 5).

### 3.9. Veterinarian’s Recommendations 

The respondents were asked to provide a single best suggestion to combat antimicrobial resistance. The suggestions overlapped in many cases, and the duplicate suggestions were removed and are categorized into field level, policy level, and research level suggestions in Supplementary Material Table S2.

## 4. Discussion

In developing countries, possible factors for antibiotic resistance include increased and indiscriminate use of antibiotics in animal production, poor farm biosecurity, inadequate infection control practices in consort with lack of compliance with regulatory frameworks [21]. In Indian dairy herds, more than 70% of production losses have been incurred by mastitis, which remains the condition requiring the most antibiotic use [22]. Similarly, in the present study, veterinarians reported mastitis as the most common condition in bovines requiring antibiotic use followed by reproductive disorders and hemoprotozoan infections.

There are limited studies from India on antibiotic usage patterns for various conditions in animal husbandry [12]. The present study listed major disease conditions of bovines requiring antibiotic usage. Our study reports the use of HPCIA in animal therapeutics, with quinolones and third-generation cephalosporins as prime antibiotics used for mastitis and metritis. However, studies from western countries reported the use of non-HPCIA predominating in animal agriculture, while the use of critically important antimicrobials was limited to the treatment of diarrhea and respiratory diseases in bovines [23]. Similarly, in Australia, the major antibiotics in bovine therapeutics were tetracycline/doxycycline, penicillin, synthetic penicillin, and trimethoprim–sulfamethoxazole [16]. In addition, the alternate systems of medicine are prevalent both in the human and veterinary sector in India [24,25,26], and the veterinarians in the study also reported the widespread usage of herbal medicines and homeopathy in bovine therapeutics.

While choosing the antibiotics, previous experience of veterinarians remained the topmost criteria, which is in accordance with previous studies where veterinarian’s prior experience of a drug was decisive for antibiotic selection [27]. Moreover, the cost of antibiotics had a moderate influence on antibiotic choice, as also reported by Australian veterinarians [16]. The lower use of antimicrobial culture and susceptibility testing in choosing antibiotics was in accordance with the study on New Zealand veterinarians [28]. The recommendations from the pharmaceutical company were a minor factor in the choice of antibiotics in contrary to the previous reports, where half of the veterinarians were influenced by the pharmaceutical companies [29].

In the present study, 69.5% of veterinarians had average knowledge score similar to earlier regional study from India, where 58.3% of veterinarians had a medium level of awareness on antibiotic resistance [30]. The majority of veterinarians had attitude in the neutral range and moderate practice scores, suggesting the need for more directed efforts on improving attitude and practices toward judicious antibiotic use. The highest knowledge and attitude scores were in the age group of <30 years and in veterinarians with <10 years of experience, which is in similar to earlier studies, where Dutch veterinarians with more years of experience were found to be less concerned about the possible contribution of veterinary antibiotic use to antimicrobial resistance [14]. The higher knowledge score among veterinarians working in veterinary polyclinics with established facilities is in accordance with reported higher social responsibility among veterinarians working in referral clinics [31]. The regional differences noted in the present study with a higher practice score for the Western region is in accordance with earlier studies where regional differences were observed [32], which might be due to the higher awareness of activities on animal husbandry practices, including farm biosecurity.

The highest consumption of antimicrobials in livestock has been reported in low- and middle-income countries where antibiotics are used for therapeutics, growth promotion, and prophylaxis [2]. In the present study, 39.0% of veterinarians reported the use of antibiotics for prophylaxis, mainly to prevent disease outbreaks, on contrary with developed nations where most veterinarians had abandoned the practice of using antibiotics for prophylaxis [33].

The reliability on diagnostic and antibiotic sensitivity testing is posited to be crucial for responsible antimicrobial use, while in the present study, 37.8% of veterinarians resorted to bacterial culture and susceptibility test results for choosing antibiotics. In addition, 70% veterinarians were not having access to well-equipped laboratory facilities for antibiotic susceptibility testing. This is in accordance with previous studies where in both veterinary [23,33] and human medicine [34], the use of antibiotic susceptibility testing for choosing antibiotics was less frequent. The lack of access to laboratory facilities for the majority of the veterinarians for confirming the root cause of treatment failure might have led to the assumption that treatment failure was due to antimicrobial resistance. Even though treatment failure may also arise due to other causes, such as the inadequate antimicrobial spectrum of the prescribed antibiotics due to the use of ineffective drugs or incorrect dosage or incorrect diagnosis, in the present study, more emphasis has been laid on antimicrobial resistance as leading causes of treatment failure, which might pose a limitation to the study.

The majority of veterinarians (87%) believed there is an ongoing antibiotic abuse in therapeutics in India, while a lower proportion of Australian livestock veterinarians opined the current usage of antibiotics as “significant” for antibiotic resistance [16]. Moreover, 98.7% veterinarians believed that antibiotic resistance was a serious public health issue, in similar line with the previous studies [35,36]. In addition, earlier studies also have reported a large number of untrained personnel (quacks) in veterinary practice in India, which might be due to unaffordable professional veterinary services for marginalized farmers [12,30,37].

The present study analyzed the possible risk factors of farmers and veterinarians for the development of treatment failure. The “illegitimate demands of farmers for antibiotic use” was significantly associated with treatment failure in accordance with the earlier studies, where around 33% veterinarians reported explicit demand of farmers for antibiotics [30]. On contrary, other study from Australia reported that the expectations of the client had a minimal influence on antibiotic prescription [16]. 

The majority of the veterinarians (82.8%) reported the purchase of antibiotics without prescription by farmers in accordance with earlier studies from India, where the lack of adequate knowledge among farmers and easy access to antibiotics without prescriptions were considered as possible drivers of this risk practice [38]. Around 31.8% of veterinarians have conducted training programs to improve knowledge of farmers on antibiotic usage. Earlier studies also reported that the majority of veterinarians believed in educating farmers on good management practices for reducing antimicrobial use [15,39]. 

In accordance with earlier studies where the Australian veterinarians have highlighted the need for cost-effective culture and susceptibility testing as well as rapid and affordable diagnostic tests for facilitating judicious antibiotic use [16], the present study has also put forward similar suggestions at the field level, regulatory level, and research level. The participating veterinarians of the present study have also emphasized the need for a data-driven interdisciplinary approach that is crucial for combating antimicrobial resistance. The present study could not have the exact proportional number of respondents from different regions of the country, which might pose a limitation. However, the study is the first of its kind to have a comprehensive approach on the existing antibiotic usage practices, KAP survey, and veterinarian’s recommendations to address antimicrobial resistance.

## 5. Conclusions

To conclude, the facilitating changes in the attitude and practices of veterinarians can be augmented by the implementation of continuing veterinary education programs. The effective flow of information from veterinarians to farmers can create a paradigm shift in the perceptions of the farmers for judicious antibiotic use as well as less reliability on quacks. There is need to strengthen the laboratory surveillance networks, research and diagnostics, and judicious antimicrobial stewardship. More stringent guidelines on the use of HPCIA in the animal sector and the compliance with responsible antimicrobial prescription behaviors by veterinarians need to be implemented. A “One Health” framework facilitating behavioural change interventions in farmers and veterinarians by bringing all the stakeholders together and promoting prudent antimicrobial use and judicious antimicrobial stewardship is the need of the hour.

## Figures and Tables

**Table 1:** Demographic information of respondents.

Characteristics	*n*	(%)
**Age (years)**		
<30	148	31.8
30–40	175	37.5
40–50	103	22.1
50–60	32	6.9
60–70	8	1.7
**Sex**		
Male	326	70.0
Female	140	30.0
**Level of education**		
Bachelor of Veterinary Sciences and Animal husbandry (B.V.Sc and A.H)	208	44.6
Master of Veterinary Sciences (M.V.Sc)	224	48.1
Ph.D.	34	7.3
**Field Experience (years)**		
<10	293	62.9
10–20	105	22.5
20–30	50	10.7
30–40	17	3.6
40–50	1	0.2
**Regional distribution (6 regions: 25 States)**		
Northern Region (Jammu and Kashmir, Haryana, Himachal Pradesh, Punjab, Delhi, Uttarakhand, Uttar Pradesh)	181	38.8
Southern Region (Andhra Pradesh, Telangana, Karnataka, Kerala, Tamil Nadu)	158	33.9
Western Region (Rajasthan, Gujarat, Maharashtra)	55	11.8
Eastern Region (Bihar, Orissa, West Bengal)	38	8.1
Central Region (Madhya Pradesh, Chhattisgarh)	22	4.7
North-East Region (Assam, Sikkim, Nagaland, Meghalaya, Mizoram)	12	2.6
**Type of Hospital**		
Veterinary Hospital (Institutes with basic facilities for day-to-day treatment and care of livestock)	399	85.6%
Veterinary Polyclinic (Institutes with specialized facilities including diagnostic laboratories)	67	14.4%

**Table 2:** Knowledge, attitude, and practices (KAP) of veterinarians regarding antibiotic use and resistance.

KAP Parameters ^¶^	Correct Answer	Percentage (%)
**Knowledge parameters**		
Is there an ongoing antibiotic abuse in therapeutics in the veterinary sector?	401	86.0
Do you know about the critically important list of antimicrobials specified by the World Health Organization (WHO)?	212	45.5
Is antibiotic resistance a serious public health issue?	460	98.7
Is antibiotic resistance a natural as well as anthropogenic phenomenon?	268	57.5
Does irrational antibiotics use in animals lead to resistance in humans?	409	87.8
Are you familiar with superbug New Delhi metallo-beta-lactamase 1?	233	50.0
Are you familiar with Livestock-associated methicillin-resistant *Staphylococcus aureus* (LA-MRSA)?	297	63.7
Does the use of expired antibiotics lead to emergence of resistance?	195	41.8
Does injudicious use of antibiotics lead to antibiotic residues in milk and meat?	450	96.6
Does antibiotic residues in milk/meat lead to emergence of resistance?	427	91.6
Are you aware about recommendations of National Antimicrobial Resistance Plan 2017 of India?	97	20.8
**Attitude parameters**		
I believe the use of two or more classes of antibiotics in combination is always a better choice to control infections	18 387 *	3.9 83.0
I believe a broad spectrum antibiotics is a better choice than using highly selective antibiotics, even when narrow-spectrum drugs are available	24	5.1
I believe priority antibiotics must be restricted for human-use only	190	40.8
I believe that skipping 1 or 2 doses of antibiotics contributes to the development of resistance	269	57.7
**Practice parameters**		
What is your first line of treatment for pyrexia of unknown origin (PUO)?	138	29.6
How often do you use bacterial culture and susceptibility testing to select the appropriate antibiotics during your treatment?	15 161 *	3.2 34.5
Illegitimate demands of farmers lead to use of antibiotics in conditions which do not require their use	163	35.0
How often do you advise the farmer to administer antibiotics through a telephonic conversation (vocal prescription)?	284	60.9
Do you write a prescription of antibiotics to farmers who come to you at the hospital without presenting their animals?	253	54.3
How often do you give free samples of antibiotics to farmers?	189	40.6
Do you use antibiotics for prophylaxis?	284	60.9
Do you check the expiry date of the antibiotics before use?	439	94.2
Do you allow the farmer to inject the subsequent doses of antibiotics after you have administered the first dose of the treatment?	292	62.7
After antibiotic treatment, do you advise farmers about not to use or sell milk up to recommended withdrawal period?	233 197 *	50.0 42.3
Do you adhere to the recommendations of the National Antimicrobial Resistance Plan of India?	48 180 *	10.3 38.6
Have you attended any trainings/conferences to update your knowledge on antibiotic usage and antimicrobial resistance?	127	27.2
Have you conducted/organized any training to improve the knowledge of farmers on antibiotic usage and antimicrobial resistance emergence?	148	31.8

* Partially correct answers. ^¶^ Each question was scored with score of 1 for correct, 0.5 for partially correct, and 0 for incorrect or not sure responses.

**Table 3:** Demographic characteristics and associated KAP scores.

Variables	Median Knowledge Score	*p*-Value *	Median Attitude Score	*p*-Value *	Median Practice Score	*p*-Value *
**Age group (years) ^¶^**						
<30	8.0	**0.03**	1.5	0.08	6.0	0.73
30–40	7.0		1.0		6.0	
40–50	7.0		1.0		5.5	
50–60	7.0		0.8		5.8	
60–70	5.5		1.3		6.8	
**Educational qualification ^¶^**						
B.V.Sc and A.H	7.0	**0.00**	1.0	0.19	6.0	0.19
M.V.Sc	8.0		1.5		6.0	
PhD	9.0		1.3		7.0	
**Years of Experience ^¶^**						
<10	8.0	**0.00**	1.5	0.24	6.0	0.33
10–20	7.0		1.0		5.5	
20–30	7.0		1.3		6.5	
30–40	5.0		0.5		5.5	
**Gender ^#^**						
Male	7.5	0.44	1.0	0.37	6.0	0.50
Female	8.0		1.5		6.0	
**Region ^¶^**						
Northern	8.0	0.12	1.5	0.09	5.5	0.02
Southern	7.0		1.5		6.3	
Central	8.0		0.5		5.5	
Western	8.0		1.0		7.0	
Eastern	8.0		1.5		5.5	
North Eastern	8.5		1.5		6.3	
**Type of hospital ^#^**						
Veterinary hospital	7.0	**0.03**	1.5	0.63	6.0	0.40
Veterinary polyclinic	8.0		1.5		6.0	

* Significant *p*-values are presented in bold characters. **^¶^** Kruskal–Wallis H test; **^#^** Mann–Whitney U test.

**Table 4:** Univariable analysis: (**a**) Veterinarians; (**b**) Farmers.

Variables	Odds Ratio (95% C.I.)	*p*-Value
(**a**)		
Use of antibiotics for prophylaxis	1.6 (1.0–2.4)	0.05
Allowing farmer to inject the subsequent doses of antibiotics after administering the first dose of treatment	1.8 (1.2–2.8)	0.009
After antibiotic treatment, advising farmers not to use or sell milk up to the recommended withdrawal period	1.1 (0.7–1.7)	0.74
Checking of expiry date of the antibiotics before use	1.9 (0.8–4.3)	0.14
Vocal prescription of antibiotics to farmers	1.5 (0.8–2.6)	0.20
Giving free samples of antibiotic to farmers	1.3 (0.7–2.2)	0.39
Skipping of 1 or 2 doses of antibiotics in the course	1.7 (1.1–2.6)	0.02
(**b**)		
Illegitimate demand of farmers for antibiotics in conditions that do not require their use	3.7 (2.3–6.0)	0.00
Farmer’s non-cooperation in completion of the antibiotic course specified by the veterinarians	1.9 (1.2–3.1)	0.007
Farmers acquiring antibiotics directly from a pharmacy without prescription	2.2 (1.1–4.4)	0.03

**Table 5:** Multivariable logistic regression analysis: (**a**) Veterinarians; (**b**) Farmers.

Variable	B	S.E	Odds Ratio (95% C.I.)	*p*-Value
(**a**)				
Skipping of 1 or 2 doses of antibiotics in the course	0.5	0.2	1.7 (1.1–2.6)	0.02
Allowing the farmer to inject the subsequent doses of antibiotics after administering the first dose of treatment	0.6	0.2	1.8 (1.1–2.8)	0.01
Constant	−1.8	0.2	0.2	0.00
Hosmer–Lemeshow test for Goodness of Fit: *p*-value = 0.93				
(**b**)				
Illegitimate demand of farmers for antibiotics in conditions that do not require their use	1.3	0.2	3.6 (2.3–5.8)	0.00
Farmer’s non-cooperation in completion of antibiotic course specified by the veterinarians	0.6	0.2	1.8 (1.1–3.0)	0.02
Constant	−2.1	0.2	0.1	0.00
Hosmer–Lemeshow test for Goodness of Fit: *p*-value = 0.55				

## Data Availability

The data presented in this study are available on request from the corresponding author. The data are not publicly available due to privacy and confidentiality agreements to the participants.

## Supplementary Materials

The following are available online at https://www.mdpi.com/2079-6382/10/2/216/s1, File S1: Questionnaire for veterinarians, Table S1: List of KAP parameters and their correct responses in questionnaire, Table S2: Recommendations at various levels from the study.
