# Supplementary material for: Knowledge, Attitude, and Practices (KAP) Survey among Veterinarians, and Risk Factors Relating to Antimicrobial Use and Treatment Failure in Dairy Herds of India
Source: Antibiotics (Basel). 2021 Feb 22;10(2):216. doi: 10.3390/antibiotics10020216 (PMC7926553; doi:10.3390/antibiotics10020216)
Supplement: Supplementary file 1 [file antibiotics-10-00216-s001.zip › Final_Supplementary Table S1.docx]

**Table S1: List of KAP parameters and their correct responses in questionnaire**

| **KAP Parameters** | **Correct Answer** | **Partially Correct Answer** |
| --- | --- | --- |
| **Knowledge parameters** | | |
| Is there an ongoing antibiotic abuse in therapeutics in veterinary sector? | Yes |  |
| Do you know about critically important list of antimicrobials specified by World Health Organization (WHO)? | Yes |  |
| Is antibiotic resistance a serious public health issue? | Yes |  |
| Is antibiotic resistance a natural as well as anthropogenic phenomenon? | Both |  |
| Does irrational antibiotics use in animals lead to resistance in humans? | Yes |  |
| Are you familiar with superbug New Delhi metallo-beta-lactamase 1? | Yes |  |
| Are you familiar with Livestock-associated methicillin-resistant *Staphylococcus aureus* (LA-MRSA)? | Yes |  |
| Does the use of expired antibiotics lead to emergence of resistance? | Yes |  |
| Does injudicious use of antibiotics lead to antibiotic residues in milk and meat? | Yes |  |
| Does antibiotic residues in milk/meat lead to emergence of resistance? | Yes |  |
| Are you aware about recommendations of ‘National Antimicrobial Resistance Plan-2017’ of India? | Yes |  |
| **Attitude parameters** | | |
| I believe use of two or more class of antibiotics in combination is always better choice to control infections | No | Sometimes |
| I believe broad spectrum antibiotics are better choice than using highly selective antibiotics, even when narrow spectrum drugs are available | No |  |
| I believe priority antibiotics must be restricted for human-use-only | Yes |  |
| I believe that skipping of 1 or 2 doses of antibiotics contributes to the development of resistance | Yes |  |
| **Practice parameters** | | |
| What is your first line of treatment for pyrexia of unknown origin (PUO)? | Antipyretics alone |  |
| How often do you use bacterial culture and susceptibility testing to select the appropriate antibiotics during your treatment? | Always | Sometimes |
| Illegitimate demands of farmers lead to use of antibiotics in conditions which don’t require their use | No |  |
| How often do you advise the farmer to administer antibiotics through telephonic conversation (vocal prescription)? | Rarely |  |
| Do you write prescription of antibiotics to farmers who come to you at hospital without presenting their animals? | No |  |
| How often do you give free samples of antibiotic to farmers? | Never |  |
| Do you use antibiotics for prophylaxis? | No |  |
| Do you check expiry date of the antibiotics before use? | Always |  |
| Do you allow the farmer to inject the subsequent doses of antibiotics after you have administered the first dose of the treatment? | No |  |
| After antibiotic treatment, do you advise farmers about not to use or sell milk up to recommended withdrawal period? | Always | Sometimes |
| Do you adhere to recommendations of National Antimicrobial Resistance Plan, India? | Always | Sometimes |
| Have you attended any trainings/conferences to update your knowledge on antibiotic usage and antimicrobial resistance? | Yes |  |
| Have you conducted/organized any training to improve the knowledge of farmers on antibiotic usage and antimicrobial resistance emergence? | Yes |  |
