# Supplementary material for: Knowledge, Attitude, and Practices (KAP) Survey among Veterinarians, and Risk Factors Relating to Antimicrobial Use and Treatment Failure in Dairy Herds of India
Source: Antibiotics (Basel). 2021 Feb 22;10(2):216. doi: 10.3390/antibiotics10020216 (PMC7926553; doi:10.3390/antibiotics10020216)
Supplement: Supplementary file 1 [file antibiotics-10-00216-s001.zip › Final_File S1_Veterinarian Questionnaire.docx]

**Part-I**

**Personal information**

**Name:_______________________ Mobile number: _____________**

**Official designation: _______________**

1. **District: ___________ State: _________**
2. **Age: ________ years**
3. **Gender: ☐ Male ☐ Female**
4. **Highest Educational qualification: ☐ B.V.Sc & A.H ☐ M.V.Sc ☐ PhD**
5. **Type of hospital in which you are working:**

**☐ Veterinary Hospital ☐ Veterinary Polyclinic**

1. **How many years of experience as a field veterinarian? ______ years**

**Part II**

**Health services**

1. **Top 03 disease conditions which require antibiotic use in bovines?**
2. **_______________**
3. **_______________**
4. **_______________**
5. **Top 03 frequently used antibiotics in treatment of bovine**

**____ 3^rd^ gen. Cephalosporins: Ceftiofur, Ceftriaxone, Cefotaxime etc.**

**____ 4^th^ gen. Cephalosporins: Cefepime, Cefquinome**

**____ 5^th^ gen. Cephalosporins: Ceftaroline**

**____ 1^st^ and 2^nd^ gen. Cephalosporins**

**____ Polymixin/Colistin**

**____ Glycopeptides: Vancomycin**

**____ Macrolides: Tylosin/Erythromycin**

**____ Quinolones: Enrofloxacin**

**____ Aminoglycosides: Gentamicin**

**____ Carbapenems: Meropenems/Imipenems**

**____ Penicillin/Streptopenicillin**

**____ Semi-Synthetic penicillins: Ampicillin/Amoxicillin**

**____ Tetracycline/Oxytetracycline**

**____ Chloramphenicol**

**____ Nitroimidazoles: Metronidazoles**

**____ Sulphonamides**

**____ Any other _______________**

1. **Top 03 frequently used antibiotics for treatment of mastitis in bovines?**

**____ 3^rd^ gen. Cephalosporins: Ceftiofur, Ceftriaxone, Cefotaxime etc.**

**____ 4^th^ gen. Cephalosporins: Cefepime, Cefquinome**

**____ 5^th^ gen. Cephalosporins: Ceftaroline**

**____ 1^st^ and 2^nd^ gen. Cephalosporins**

**____ Polymixin/Colistin**

**____ Glycopeptides: Vancomycin**

**____ Macrolides: Tylosin/Erythromycin**

**____ Quinolones: Enrofloxacin**

**____ Aminoglycosides: Gentamicin**

**____ Carbapenems: Meropenems/Imipenems**

**____ Penicillin/Streptopenicillin**

**____ Semi-Synthetic penicillins: Ampicillin/Amoxicillin**

**____ Tetracycline/Oxytetracycline**

**____ Chloramphenicol**

**____ Nitroimidazoles: Metronidazoles**

**____ Sulphonamides**

**____ Any other _______________**

1. **Top 03 frequently used antibiotics for the treatment of metritis in bovines?**

**____ 3^rd^ gen. Cephalosporins: Ceftiofur, Ceftriaxone, Cefotaxime etc.**

**____ 4^th^ gen. Cephalosporins: Cefepime, Cefquinome**

**____ 5^th^ gen. Cephalosporins: Ceftaroline**

**____ 1^st^ and 2^nd^ gen. Cephalosporins**

**____ Polymixin/Colistin**

**____ Glycopeptides: Vancomycin**

**____ Macrolides: Tylosin/Erythromycin**

**____ Quinolones: Enrofloxacin**

**____ Aminoglycosides: Gentamicin**

**____ Carbapenems: Meropenems/Imipenems**

**____ Penicillin/Streptopenicillin**

**____ Semi-Synthetic penicillins: Ampicillin/Amoxicillin**

**____ Tetracycline/Oxytetracycline**

**____ Chloramphenicol**

**____ Nitroimidazoles: Metronidazoles**

**____ Sulphonamides**

**____ Any other _______________**

1. **What is your first line of treatment for pyrexia of unknown origin (PUO)?**

**☐ Antibiotics + Antipyretics**

**☐ Antibiotics alone**

**☐ Antipyretics alone**

**☐ Any other, please specify ______________________**

1. **What are the top 03 factors in determining the choice of antibiotics use in your treatment? Choose among the following**

**(Own experience/Availability of antibiotic(s)/Recommendations from other veterinarians/Cost of antibiotic /Positive culture and susceptibility tests/Drug withdrawal times/Recommendations from pharmaceutical company)**

1. **________________________**
2. **________________________**
3. **________________________**
4. **How often do you use bacterial culture and susceptibility testing to select the most appropriate antibiotics during your treatment?**

**☐ Always ☐ Sometimes ☐ Rarely**

1. **Is there well-equipped laboratory facility for performing antibiotic susceptibility testing (ABST) available in/near your hospital?**

**☐ Yes ☐ No**

**If Yes, Name the facility ________________________**

1. **How often do you advise the farmer to administer antibiotics through telephonic conversation?**

**☐ Rarely ☐ Frequently**

1. **Do you write prescription of antibiotics to farmers who come to you at hospital without presenting their animals?**

**☐ Yes ☐ No**

1. **How often do you give free samples of antibiotic to farmers?**

**☐ Never ☐ Sometimes ☐ Frequently**

1. **Do you use antibiotics for prophylaxis?**

**☐ Yes ☐ No**

**If yes, please mention the conditions of usage? ________________**

1. **Do you check expiry date of the antibiotic before using?**

**☐ Always ☐ Sometimes ☐ Never**

1. **Do farmers cooperate in the completion of antibiotic course specified by you?**

**☐ Yes ☐ No**

1. **Do you ask the farmer to administer the subsequent doses of antibiotics after you have administered the first dose of treatment?**

**☐ Yes**  **☐ No**

**Part III**

**Antibiotics**

1. **I believe that use of two or more class of antibiotics in combination is always a better choice to control infections**

**☐ Yes ☐ Sometimes ☐ No**

1. **I believe broad spectrum antibiotics are better choice than using highly selective antibiotics even when narrow spectrum drugs are available**

**☐ Yes ☐ No**

1. **Name top 03 pharmaceutical companies whose antibiotic you use frequently**
2. **________________________**
3. **________________________**
4. **________________________**
5. **Is there an ongoing antibiotic abuse in therapeutics in veterinary sector?**

**☐ Yes ☐ No**

1. **Do you know about critically important list of antimicrobials specified by World Health Organization (WHO)?**

**☐ Yes ☐ No**

1. **What is your opinion on restricting ‘priority antibiotics’ for human-use-only?**

**☐ Yes, we are avoiding antibiotics for ‘human-use-only’ in animals**

**☐ No, it’s not possible to completely avoid these drugs**

1. **Illegitimate demand of farmers leads to use of antibiotics in conditions which do not require their use?**

**☐ Yes, the demand affect use ☐ No, the demand doesn’t affect use**

1. **I have experienced that farmers are acquiring antibiotics directly from a pharmacy without having prescription from a veterinary doctor**

**☐ Yes ☐ No**

**Part-IV**

**Antimicrobial resistance**

1. **Whom do you think as responsible for irrational use of antibiotic in bovines at field level (select all that apply)?**

**☐ Veterinarian ☐ Para-veterinarian ☐ Class IV**

**☐ Quack ☐ Farmers**

1. **How frequent have you found a course of antibiotic was ineffective for animals’ treatment in the past one year?**

**☐ Rarely ☐ Frequently**

1. **Based on your experience, rank the disease conditions and associated antibiotics found to be ineffective.**

**Disease/condition Treatment schedule**

1. **______________ _____________________**
2. **______________ _____________________**
3. **______________ _____________________**
4. **______________ _____________________**
5. **______________ _____________________**

**☐ I didn’t notice any resistance for on-going treatment schedules**

1. **Is antibiotic resistance a serious public health issue?**

**☐ Yes, it is a serious issue ☐ No, It’s not a serious issue**

1. **Is antibiotic resistance a natural as well as anthropogenic phenomenon?**

**☐ Natural only ☐ Anthropogenic only ☐ Both ☐ Not Sure**

1. **Does irrational antibiotic use in animals lead to antibiotic resistance in humans?**

**☐ Yes ☐ No ☐ Not Sure**

1. **Are you familiar with superbug New Delhi metallo-beta-lactamase 1?**

**☐ Yes ☐ No**

1. **Are you familiar with superbug Livestock-associated methicillin-resistant *Staphylococcus aureus* (LA-MRSA)?**

**☐ Yes ☐ No**

1. **I believe that skipping of 1 or 2 doses of antibiotics contribute to the development of antibiotic resistance**

**☐ Yes ☐ No ☐ Not Sure**

1. **I believe ‘priority antibiotics’ must be restricted for human-use-only**

**☐ Yes ☐ No**

1. **Does the use of expired antibiotics lead to emergence of resistance?**

**☐ Yes ☐ No ☐ Not Sure**

1. **After antibiotic treatment, do you advise farmers about not to use or sell milk up to recommended withdrawal period?**

**☐ Always ☐ Sometimes ☐ Never**

1. **Does injudicious use of antibiotics lead to antibiotic residues in milk and meat?**

**☐ Yes ☐ No ☐ Not Sure**

1. **Does antibiotic residues in milk/meat lead to emergence of resistance?**

**☐ Yes ☐ No ☐ Not Sure**

**Part V**

**Miscellaneous**

1. **Please share other practices or products besides antibiotics that you use to prevent or treat disease(s) [select all that apply]**

**☐ Homeopathic medicines ☐ Herbal medicines**

**☐ Probiotics ☐ No other option available**

**☐ Indigenous remedies**

1. **If applicable, mention the alternate products used for the following**

**Disease/condition Treatment schedule**

1. **Mastitis _____________________**
2. **Metritis _____________________**
3. **Retention of Placenta _____________________**
4. **Pneumonia _____________________**
5. **Diarrhoea _____________________**
6. **Please mention the drugs other than antibiotics (e.g.: anti-parasitic, antifungal etc.) found to be resistant during treatment**

**Disease/condition Treatment schedule**

1. **______________ _____________________**
2. **______________ _____________________**
3. **______________ _____________________**
4. **______________ _____________________**
5. **______________ _____________________**

**☐ Didn’t notice any resistance for drugs other than antibiotics**

1. **Are you aware about the recommendations of National Antimicrobial Resistance Plan-2017 of India?**

**☐ Yes ☐ No**

1. **If yes in previous question, do you adhere to the recommendations of National Antimicrobial Resistance Plan-2017, India?**

**☐ Not possible in field ☐ Sometimes ☐ Always**

1. **Have you attended any trainings/conferences to update your knowledge on antibiotic usage and antimicrobial resistance?**

**☐ Yes ☐ No**

**If yes, details of training ________________________________**

1. **Have you conducted/organized any training to improve the knowledge of farmers on antibiotic usage and antimicrobial resistance emergence?**

**☐ Yes ☐ No**

1. **What are the major information sources that you refer regularly for the knowledge on antibiotic use and resistance?**
2. **_____________**
3. **_____________**
4. **_____________**

**☐ Not referring any source**

1. **What would be the best single suggestion that you recommend to the policy makers to combat antimicrobial resistance?**

**________________________________________________________________**

**________________________________________________________________**

**-******* THE END **********
