# Supplementary material for: Knowledge, Attitude, and Practices (KAP) Survey among Veterinarians, and Risk Factors Relating to Antimicrobial Use and Treatment Failure in Dairy Herds of India
Source: Antibiotics (Basel). 2021 Feb 22;10(2):216. doi: 10.3390/antibiotics10020216 (PMC7926553; doi:10.3390/antibiotics10020216)
Supplement: Supplementary file 1 [file antibiotics-10-00216-s001.zip › Supplementary Table S2.docx]

**Table S2: Recommendations at various levels from the study**

| **Field Level** | **Regulatory Level** | **Research Level** |
| --- | --- | --- |
| 1. Rational use of antibiotics after proper disease diagnosis. 2. Avoidance of multiple antibiotics therapy or combination therapy when narrow spectrum alternatives are available. 3. Avoid antibiotics that are critically important for human medicine. 4. Avoidance of use of higher generation antibiotics as first line of treatment. 5. Avoidance of use of antibiotics showing high resistance for specific disease conditions in a given area. 6. Ensure that antibiotics should be administered only by registered practitioners. 7. Veterinarians should ensure no use of antibiotics in prophylaxis or growth promotion. 8. Motivate farmers to adhere to complete antibiotic course. 9. Use of alternative therapies (ethnoveterinary practice) should be advocated. 10. Cost-effective diagnostic facilities should be provided to veterinarians at field level so that they can be specific with available treatment options. 11. Mass awareness for judicious use of antibiotics should be created among all stakeholders including veterinarians, para-veterinarians, farmers etc. 12. Encourage vaccination for the vaccine preventable diseases. 13. Promote farm biosecurity measures and infection control practices at farm level. 14. Educate public about health hazards of antibiotics resistance. | 1. Strict enforcement of veterinary drug dispensing only on the prescription of registered veterinarians. 2. Strict guidelines against the use of antibiotics other than therapeutics. 3. Censoring on unauthorized veterinary practice by quacks. 4. Strict compliance on withdrawal periods of antibiotics and provide compensation to farmers for the losses. 5. Strengthen the quality control units to avoid marketing of sub-standards antibiotics. 6. Sale of drugs by pharmacists should be regularly audited by drug authorities. 7. Increase the strength of veterinarians in the field to avoid unauthorized animal health services. 8. Strengthening of the network of antibiotic sensitivity testing laboratories. 9. Regulations in favour of the sale of antibiotics as a complete course. 10. Regular testing of milk samples for antibiotic residues at milk collection centres and provide incentives to farmers having no drug residues in their products. 11. The government should provide subsidies to marginalized farmers on antibiotic therapy so that they can be encouraged to complete the course. 12. Establishment of national as well as regional database for surveillance of antibiotics usage in animal health sectors. 13. Continuing veterinary education programs for field veterinarians. 14. Strengthening of ‘one-health’ framework to address the antimicrobial resistance issue in more effective manner. | 1. Development of rapid and cost-effective diagnostics to identify drug resistant bacteria at field level. 2. Research on alternate therapies (e.g., bacteriophages, antimicrobial peptides, herbal drugs etc) should be promoted. 3. Development of effective vaccines for the endemic diseases. 4. Effective monitoring and tracing of antibiotic resistance genes. 5. Using antibiotic resistance surveillance data to make out the list of ‘to-use’ and ‘not-to-use’ antibiotics for particular disease conditions in specific region. 6. Development of cost-effective quality antibiotics for field use. 7. Development of training modules persuading the veterinarians and farmers for prudent antibiotic use. 8. Regular publication of magazine or periodicals to update the veterinarians and farmers about antibiotic related issues in regional languages. |
